# Green endoscopy: Economic and ecological evaluation of single‐use versus reusable ureterorenoscopes

**DOI:** 10.1002/bco2.70100

**Published:** 2025-10-08

**Authors:** Marcel Schwinger, Charis Kalogirou

**Affiliations:** ^1^ Department of Urology and Pediatric Urology Julius Maximilians University Medical Center of Würzburg Würzburg Germany

**Keywords:** carbon footprint, economics, single‐use medical devices, sustainability, ureterorenoscopy

## Abstract

**Objective:**

The number of ureterorenoscopies in Germany is rising. Hospitals must operate economically while ensuring quality. Environmental assessment of medical procedures is gaining focus. This study aims to perform a comparative analysis of the economic and ecological aspects of single‐use versus reusable ureterorenoscopes using real‐world routine data, acknowledging their trade‐offs between hygienic advantages, costs and environmental impacts.

**Materials and Methods:**

A total of 210 ureterorenoscopy cases (2022/2023) at the University Hospital of Würzburg were evaluated. A simulation assessed the impact of the OPS code 5‐98b.0 on DRG (diagnosis‐related groups) classification and reimbursement. Economic analysis included acquisition, repair and sterilization costs, while ecological assessment considered manufacturing, waste, reprocessing, transport and repair.

**Results:**

In 44.3% of cases, use of OPS code 5‐98b.0 resulted in an upgraded DRG (L20B instead of L20C), yielding approximately €62 000 in additional revenue over 2 years. This was outweighed by roughly €147 000 in extra costs for single‐use devices, assuming repair costs for reusable devices remained around €300 per case. Environmentally, single‐use devices generated 42 kg more CO_2_ per 100 procedures.

**Conclusion:**

Single‐use ureterorenoscopes are economically justifiable only when reusable devices incur frequent repair costs. Reusable scopes perform better ecologically due to lower CO_2_ emissions. Instrument choice should be guided by each clinic's specific economic and environmental context.

## 1 INTRODUCTION

Ureterorenoscopy (URS) represents one of the most frequently performed minimally invasive endourological procedures, with over 78 000 interventions per year in the inpatient sector in Germany alone. The annual number of URS procedures more than doubled from 32 203 in 2006 to 78 125 in 2019.^1^ URS is primarily employed for the diagnosis and treatment of ureteral and renal stones as well as urothelial carcinomas of the upper urinary tract. Thanks to technical advancements—particularly the introduction of flexible and increasingly miniaturized ureterorenoscopes—the scope of application for this technique has broadened substantially over the past two decades. In parallel, awareness of the financial and environmental impacts of the instruments used has intensified.

The growing adoption of single‐use endoscopes in clinical practice, including urology, raises new questions regarding their economic viability and sustainability compared to conventional reusable endoscopes. While companies chiefly promote reduced repair costs, simplified workflows and potential DRG advantages, these benefits are counterbalanced by substantial acquisition costs and a potentially significant increase in plastic waste and CO_2_ emissions. It must also be noted that many single‐use instruments are manufactured outside Europe and transported over long distances.^2^, ^3^ In addition to the associated transport emissions, the economic calculations may be influenced by comparatively low labour costs in the countries of origin, raising further considerations regarding ethical aspects of worker treatment and supply chain transparency.

Against this background, a comprehensive comparison of single‐use versus reusable ureterorenoscopy from both economic and ecological perspectives is warranted. This study extends the existing literature by jointly evaluating the economic and ecological aspects of single‐use versus reusable ureterorenoscopes based on real‐world routine data and DRG‐coded reimbursement information from a German university hospital, thereby providing context‐specific insights that go beyond previously published micro‐costing and environmental modelling studies.

The aim of the present study was to perform a model‐based evaluation of single‐use versus reusable ureterorenoscopes using real‐world routine data from a German university hospital, integrating a revenue simulation based on the German Diagnosis Related Groups (DRG) system and a standardized carbon footprint assessment.

## 2 MATERIALS AND METHODS

For the economic assessment, a retrospective simulation was conducted based on 210 URS cases at the University Hospital Würzburg (2022/2023). Procedures coded under the Operations and Procedures Classification System (OPS) as reusable ureterorenoscopes (OPS 5‐98b.x) were virtually replaced by the single‐use URS device code (OPS 5‐98b.0), and the resulting DRG assignments were analysed using the ID DIACOS Grouper, a validated software tool for automated DRG grouping in the German hospital billing system. In the economic analysis, acquisition and repair costs of reusable ureterorenoscopes were included, as well as sterilization costs associated with their reprocessing. Personnel and training costs were not explicitly modelled, as they were assumed to be comparable between single‐use and reusable systems and thus not expected to influence the differential cost analysis.

For the CO_2_ calculation, the analysis included emissions related to manufacturing, waste management, sterilization, transport, reprocessing and repair. Emissions were modelled in accordance with the methodology described by Davis et al., which links procedure‐specific resource consumption to published emission factors for medical devices and energy use. For each ureterorenoscopy, material inputs (e.g., single‐use instruments, packaging, cleaning agents) and energy requirements (e.g., reprocessing of reusable devices, waste disposal) were quantified based on a targeted literature review and additional information obtained from manufacturers. These inputs were then converted into CO_2_ equivalents using the CO_2_ calculator developed by Henniger et al., which applies standardized conversion factors derived from internationally recognized life‐cycle assessment (LCA) data. This approach enables a consistent and transparent estimation of the carbon footprint associated with both single‐use and reusable ureterorenoscopes.^3^, ^4^

No formal sensitivity analysis or uncertainty range was performed for the CO_2_ estimates.

## 3 RESULTS

The DRG simulation revealed that the single‐use device code (OPS 5‐98b.0) led to assignment to a higher valued DRG (L20B instead of L20C) in 44.3% of cases, resulting in additional revenues exceeding €62 000 for the two‐year period. However, acquisition costs for the single‐use instruments amounted to approximately €147 000. Repair and maintenance costs for reusable devices ranged from €153 to €512 per procedure. At a threshold of approximately €346 per case, the use of single‐use instruments becomes economically advantageous. This threshold calculation was based on the differential between additional DRG revenues generated by single‐use instruments and their higher acquisition costs compared with reusable devices. Specifically, the average repair and maintenance cost per procedure for reusable devices was iteratively varied within the observed range (€153–€512). The break‐even point of €346 per case represents the level at which the incremental DRG revenues of single‐use ureterorenoscopes offset their additional acquisition costs, such that total costs between both systems converge.

The ecological model estimated CO_2_ emissions of 177.5 kg per 100 interventions with single‐use ureterorenoscopes, compared to 135.3 kg for reusable instruments. This corresponds to an additional 42 kg of CO_2_ emissions for single‐use devices compared with reusable devices. Detailed data on the composition and carbon footprint of single‐use versus reusable URS devices are presented in Tables 1, 2, 3.

**TABLE 1:** Components and manufacturing costs of single‐use ureterorenoscopes per kg.

Material	CO_2_ equivalent mass per kg of material	% content in single‐use URS	CO_2_ footprint (kg CO_2_ per kg of device)
Plastic	6	90	5.4
Rubber	1.16	2	0.02
Metal	18	4	0.07
Electronics	150	4	6
Total	N/A	100%	11.49

**TABLE 2:** Components and manufacturing costs of reusable ureterorenoscopes per kg.

Material	CO_2_ equivalent mass per kg of material	% content in reusable‐use URS	CO_2_ footprint (kg CO_2_ per kg of device)
Plastic	6	10	0.6
Rubber	1.16	25	0.275
Metal	1.8	60	1.08
Electronics	150	5	7.95
Total	N/A	100%	9.905

**TABLE 3:** Carbon footprint per 100 procedures for single‐use (above) and reusable (below) ureterorenoscopes.

Process	CO_2_ footprint (kg CO_2_ per use)
Single‐use ureterorenoscope	
Manufacturing (device weight 0.3 kg)	103.41
Waste generation	30
Sterilization	30
Transport (30 kg from East Asia to Europe)	14.1
Total	**177.51 kg CO**_**2**_
Reusable ureterorenoscope	
Manufacturing (device weight 4 kg)	39.62
Sterilization (100× plasma sterilization)	45
Repackaging	6.5
Repair costs (5 kg CO_2_ per repair)	40
Waste generation (4 kg)	4
Transport (4 kg within Europe)	0.148
Total	**135.268 kg CO**_**2**_

## 4 DISCUSSION

This study demonstrates that the choice between single‐use and reusable ureterorenoscopes requires a nuanced evaluation. The economic analysis indicates that single‐use devices may be financially sensible in settings with high repair costs, limited sterilization capacity, or low case volumes. In particular, when frequent instrument replacement is necessary or no central reprocessing unit is available, single‐use devices can optimize workflow. Conversely, reusable instruments generally remain more cost‐effective when repair and maintenance costs are low and device utilization is sufficient.^5^ Our findings are consistent with the recent analysis by Hogan et al., who also highlighted that the economic favourability of single‐use instruments is highly context‐dependent, with repair frequency and institutional infrastructure being decisive factors.^6^

Repair susceptibility is a critical parameter: studies by Kramolowsky et al. and Sooriakumaran et al. emphasize that the cost of reusable instruments is heavily influenced by repair expenses.^7^, ^8^ In our analysis, single‐use devices were only economically justifiable when repair costs per case exceeded approximately €346, suggesting that targeted training for surgical staff and the selection of more robust instruments can contribute to cost reduction.^9^ Hogan et al. similarly reported that staff experience and adherence to reprocessing protocols significantly influence the overall cost trajectory, underlining the importance of local practices in determining cost‐effectiveness.^6^

An important finding of this study is that procedures coded with the OPS code for single‐use ureterorenoscopes were more frequently assigned to a higher‐valued DRG. This effect appears to result from the coding logic of the German DRG system, which groups cases according to resource intensity and clinical complexity rather than ecological considerations. The assignment of a higher‐valued DRG based on device category may suggest that single‐use scopes are implicitly assumed to increase procedural costs; however, the additional reimbursement only partially offsets the higher acquisition costs and is therefore unlikely to represent a deliberate incentive to promote disposable technologies. Rather, it seems more plausible that this outcome is an unintended consequence of the coding structure. Nevertheless, this raises an important policy question: given the environmental disadvantages of single‐use devices, continuous evaluation of coding frameworks is warranted to ensure better alignment between reimbursement structures, clinical practice and sustainability goals.

From an ecological standpoint, the use of single‐use ureterorenoscopes is associated with higher CO_2_ emissions and increased waste. Although literature in this field remains limited, our model indicated an excess CO_2_ output of approximately 42 kg per 100 procedures for single‐use instruments.^3^ Notably, the energy‐intensive production of plastic components and international transport—primarily from Asia—are key factors. Hogan et al. also underscored the environmental disadvantages of disposable devices, reporting comparable increases in carbon footprint relative to reusable systems.^6^ The lack of transparent life‐cycle assessments from many manufacturers further complicates standardized evaluation.

A limitation of our analysis is that break‐even costs were calculated on a per‐procedure basis and did not explicitly account for case volume. In practice, the cost‐effectiveness of reusable ureterorenoscopes improves with higher procedural volumes, as fixed acquisition and maintenance costs are distributed across a larger number of cases. By contrast, the costs of single‐use devices remain constant per intervention, independent of volume. Future studies should therefore integrate procedural volume into the modelling framework to provide more differentiated recommendations for institutions of varying size and case load.

For centres relying on single‐use ureterorenoscopes, mitigation strategies to offset the associated carbon footprint should be considered. Potential approaches include institutional participation in certified carbon offset programs, optimization of supply chains to reduce transport emissions and investment in hospital‐level sustainability initiatives such as renewable energy use or waste reduction strategies.

Improved waste disposal practices may also help reduce the ecological burden of single‐use instruments. Strategies include the segregation of medical plastics to facilitate recycling, collaboration with specialized waste management providers and the development of hospital policies that prioritize material recovery wherever safe and feasible.

Regulatory considerations also play a role: with the EU Green Deal and rising environmental reporting requirements in healthcare, legislative frameworks may soon render single‐use materials less attractive.^10^ Hospitals are therefore increasingly called upon to implement sustainability strategies that balance ecological and economic criteria.

Hygienic aspects merit discussion as well. Single‐use ureterorenoscopes inherently eliminate the risk of cross‐contamination between patients and are therefore often considered advantageous from an infection control perspective. Indeed, disposable ureteroscopes have been associated with significantly lower postoperative infection rates in comparative studies.^11^, ^12^ On the other hand, several investigations highlight that modern reprocessing protocols for reusable devices, when properly executed and combined with strict process monitoring, training and quality assurance, can achieve similarly high safety standards and very low contamination rates.^13^, ^14^ Residual contamination is typically linked to lapses in reprocessing compliance rather than to intrinsic limitations of reusable systems. Consequently, while single‐use instruments provide a theoretical hygienic advantage, the actual risk of cross‐contamination under rigorous reprocessing conditions appears minimal.

Another limitation of this study is that no formal sensitivity analysis or uncertainty range was conducted for the CO_2_ estimates. Input variability (e.g., in material composition, transport distances, or energy consumption) may therefore affect the precision of the reported values. Future studies with more granular primary data should incorporate sensitivity analyses to better capture this uncertainty.

In summary, single‐use ureterorenoscopes may present a viable alternative under specific conditions—such as high repair frequency or limited reprocessing capability—whereas reusable instruments are ecologically preferable in most scenarios. A generalized recommendation in favour of one approach is not feasible; rather, each institution must conduct an individualized analysis incorporating medical, organizational and environmental parameters.

## 5 CONCLUSION

The decision between single‐use and reusable ureterorenoscopes should be based on each hospital's specific circumstances. In training centres or facilities with restricted reprocessing capabilities, partial adoption of single‐use devices is advisable to prevent downtime caused by reusable instrument failures. Conversely, hospitals with advanced urological expertise and adequate sterilization resources should primarily employ reusable instruments, as these offer substantial cost savings and reduced CO_2_ emissions while ensuring consistent throughput. Urological sustainability strategies must increasingly integrate ecological considerations alongside economic factors.

## AUTHOR CONTRIBUTIONS

Marcel Schwinger and Charis Kalogirou conceived of the presented idea. Charis Kalogirou raised data. Marcel Schwinger performed the computations. Marcel Schwinger and Charis Kalogirou discussed the results and contributed to the final manuscript.

## CONFLICT OF INTEREST STATEMENT

The authors have no conflicts of interest to declare.

## Data Availability

All data generated or analysed during this study are included in this article. Further enquiries can be directed to the corresponding author.
